# Insights into the function of ESCRT complex and LBPA in ASFV infection

**DOI:** 10.3389/fcimb.2023.1163569

**Published:** 2023-12-06

**Authors:** Lucía Barrado-Gil, Isabel García-Dorival, Inmaculada Galindo, Covadonga Alonso, Miguel Ángel Cuesta-Geijo

**Affiliations:** Departmento Biotecnología, INIA-CSIC, Centro Nacional Instituto Nacional de Investigación y Tecnología Agraria y Alimentaria, Madrid, Spain

**Keywords:** ESCRT, LBPA, Alix, Vps4, multivesicular bodies, NPC1, ASFV, African swine fever virus

## Abstract

The African swine fever virus (ASFV) is strongly dependent on an intact endocytic pathway and a certain cellular membrane remodeling for infection, possibly regulated by the endosomal sorting complexes required for transport (ESCRT). The ESCRT machinery is mainly involved in the coordination of membrane dynamics; hence, several viruses exploit this complex and its accessory proteins VPS4 and ALIX for their own benefit. In this work, we found that shRNA-mediated knockdown of VPS4A decreased ASFV replication and viral titers, and this silencing resulted in an enhanced expression of ESCRT-0 component HRS. ASFV infection slightly increased HRS expression but not under VPS4A depletion conditions. Interestingly, VPS4A silencing did not have an impact on ALIX expression, which was significantly overexpressed upon ASFV infection. Further analysis revealed that ALIX silencing impaired ASFV infection at late stages of the viral cycle, including replication and viral production. In addition to ESCRT, the accessory protein ALIX is involved in endosomal membrane dynamics in a lysobisphosphatydic acid (LBPA) and Ca^2+^-dependent manner, which is relevant for intraluminal vesicle (ILV) biogenesis and endosomal homeostasis. Moreover, LBPA interacts with NPC2 and/or ALIX to regulate cellular cholesterol traffic, and would affect ASFV infection. Thus, we show that LBPA blocking impacted ASFV infection at both early and late infection, suggesting a function for this unconventional phospholipid in the ASFV viral cycle. Here, we found for the first time that silencing of VPS4A and ALIX affects the infection later on, and blocking LBPA function reduces ASFV infectivity at early and later stages of the viral cycle, while ALIX was overexpressed upon infection. These data suggested the relevance of ESCRT-related proteins in ASFV infection.

## 1 Introduction

Currently, African swine fever (ASF) is considered as a major emerging threat since it has increased its incidence and spread over Central and Eastern Europe to Southeast Asia, America, and Oceania, causing high economic burden and impact on animal health (Alonso et al., 2018; Mighell and Ward, 2021; Schambow et al., 2022). ASF usually results in hemorrhagic fever in domestic pigs and wild boar, with a case fatality rate close to 100%. The etiological agent causing ASF is the African swine fever virus (ASFV), the only member of the *Asfarviridae* family and also part of the nucleocytoplasmic large DNA virus (NCLDV) group, including *Mimiviridae*, *Poxviridae*, *Iridoviridae*, faustoviruses, pandoraviruses, and pacmanviruses (Andreani et al., 2017; Koonin and Yutin, 2019; Koonin et al., 2020). The development of strategies to prevent and control ASFV is a major challenge due to the dearth of commercial vaccines or antiviral treatments as well as the existence of wildlife reservoirs contributing to its impact and increasing difficulties to control the disease. Subsequently, animal movement restrictions and slaughtering of infected animals are the only possible solutions during the outbreaks (Costard et al., 2009; Urbano and Ferreira, 2022). In this scenario, it is necessary to continue to decipher the mechanisms underlying ASFV infection.

ASF virions are icosahedral multi-layered structures of approximately 200 nm in diameter, comprising a DNA-containing nucleoid, which is then surrounded by the core shell or matrix. In the internal face of the inner viral membrane, the capsid is assembled around this internal membrane. Lastly, a further envelope is acquired when the virus buds out at the host cell plasma membrane. The external envelope is not essential for viral infection. New details have been recently unveiled about the complexity of the virion structure (Liu et al., 2019; Wang et al., 2019; Andres et al., 2020).

ASFV enters the cell via clathrin- and dynamin-mediated endocytosis (Hernaez and Alonso, 2010; Galindo et al., 2015) and macropinocytosis (Sanchez et al., 2012; Hernaez et al., 2016). The late endosome is a crucial compartment for viral fusion, involving the NPC intracellular cholesterol transporter 1 (NPC1) (Matamoros et al., 2020; Cuesta-Geijo et al., 2022). Fusion would allow penetration towards the cytosol to finally start replication (Cuesta-Geijo et al., 2012; Sanchez et al., 2017). The viral factories made by ASFV are highly complex structures, induced by profound reorganization of the organelles that comprise membranes recruited from the endosomal pathway (Cuesta-Geijo et al., 2017) and the secretory pathway, which may contribute to the induction of cellular stress responses. ASFV mature particles utilize the cytoskeletal network to egress from the viral factory to the cell surface (Carvalho et al., 1988; Jouvenet et al., 2004), a travel depending on the late structural protein E120R (Andres et al., 2001).

The endosomal sorting complex required for transport (ESCRT) machinery is crucial for various cellular membrane-reorganization events including membrane remodeling, sealing, or repair, as required in several viral infections (Vietri et al., 2020; Olmos, 2022). In fact, enveloped retroviruses (HIV) and +strand RNA viruses (such as filo-, arena-, radon-, and paramyxoviruses) redirect cellular ESCRT proteins to the plasma membrane, leading to budding and fission of the viral particles from infected cells (McCullough et al., 2013; Vietri et al., 2020). In addition, the ESCRT machinery leads the constitution of the viral replication complex of a variety of viral families that require alterations of cellular membranes for successful replication (Torii et al., 2020; Meng and Lever, 2021). A closely related virus, such as Vaccinia virus (VACV), hijacks ESCRT to facilitate virus maturation, egress, and spread by ESCRT-mediated VACV wrapping. Moreover, multivesicular bodies (MVBs) serve as a major non-cisternae membrane source for the formation of intracellular enveloped viruses (IEVs) (Huttunen et al., 2021).

The ESCRT complex comprises four core complexes called ESCRT-0, ESCRT-I, ESCRT-II, and ESCRT-III, plus the vacuolar protein sorting-associated protein 4 (VPS4) and additional accessory proteins such as ALG-2 interacting protein X (ALIX) homodimer (Katzmann et al., 2001; Babst et al., 2002; Saksena et al., 2009; Schoneberg et al., 2017). ESCRT subunits are activated as a cascade, starting with ESCRT-0 (HRS) that recruits ESCRT-I, while, in turn, ESCRT-I interacts with the ESCRT-II complex. Then, ESCRT-II initiates the formation of the ESCRT-III complex that needs energy to depolymerize (Korbei, 2022; Olmos, 2022). ATPase VPS4, which has the isoforms VPS4A and VPS4B, hydrolyzes ATP to provide the necessary energy to recycle ESCRT-III (Babst et al., 1998). VPS4 balance out cytosolic ESCRT-III and allows a change of ESCRT-III filaments during the membrane scission process (Adell et al., 2014; Mierzwa et al., 2017; McCullough et al., 2018).

As mentioned before, ALIX is a crucial actor in ESCRT dynamics. It is a cytosolic protein identified on the basis of its association with pro-apoptotic signaling (Vito et al., 1999). However, ALIX regulates other cellular mechanisms, including endocytic membrane trafficking, cell adhesion, and cytoskeletal remodeling (Odorizzi, 2006). In addition, several viral families exploit ALIX for budding (Carlton et al., 2008; Zhai et al., 2011; Votteler and Sundquist, 2013) or replication (Fujii et al., 2009; Liu et al., 2022). This ability to participate in a variety of activities relies in its domain architecture, which allows ALIX to interact with the ESCRT-I subunit TSG101 (Strack et al., 2003; von Schwedler et al., 2003), and the ESCRT-III subunit CHMP4 (Odorizzi, 2006; Fisher et al., 2007; Usami et al., 2007).

ALIX tightly collaborates with LBPA, an unconventional and specific phospholipid localized primarily at the inner late endosome/lysosome (LE/LY) membranes, being crucial for intraluminal vesicle (ILV) formation in a Ca^2+^-dependent manner (Bissig et al., 2013). Moreover, lysobisphosphatydic acid (LBPA) is involved in the sorting and efflux of LE/LY components, including cholesterol intracellular traffic (Kobayashi et al., 1998; Kobayashi et al., 1999; Gruenberg, 2003; Mobius et al., 2003; Matsuo et al., 2004; Hullin-Matsuda et al., 2007). An obligate direct interaction with the cholesterol transporter NPC2 has been recently revealed, establishing the essential functional nature of NPC2–LBPA interactions in the egress of cholesterol from the LE/LY compartment. Of note is that the importance of the host cholesterol pools in viral fusion and replication including ASFV has been described (Ilnytska et al., 2013; Cuesta-Geijo et al., 2016; McCauliff et al., 2019; Ballout et al., 2020).

Our study gives indications suggesting that these protein machineries affecting cellular membrane dynamics could have a role in the ASFV infection cycle, unveiling lines of evidence about the relevance of ESCRT machinery-related proteins VPS4A and ALIX, as well as the LBPA lipid, as potential new therapeutical targets to combat the infection.

## 2 Materials and methods

### 2.1 Cell culture

The Vero (ATCC CCL-81; renal fibroblasts) cell line was cultured in Dulbecco’s modified Eagle medium (DMEM) 1% penicillin-streptomycin (P/S), and 2 mM GlutaMAX (Gibco, Gaithersburg, MD, USA) and supplemented with 5% heat-inactivated fetal bovine serum (FBS). FBS was reduced to 2% in the inoculum at the time of viral adsorption and throughout the infection process. All these mammalian cells were grown at 37°C and 5% CO2 conditions.

### 2.2 Viruses and infection

We used the Vero-adapted and non-pathogenic ASFV isolate Ba71V (Enjuanes et al., 1976) and fluorescent recombinant viruses Ba71V-30GFP (BPP30GFP) (Barrado-Gil et al., 2017) and Ba71V-Bp54GFP (B54GFP) (Hernaez et al., 2006). BPP30GFP expresses the GFP gene under the promoter of the early viral p30 protein (Barrado-Gil et al., 2017). Recombinant B54GFP expresses GFP as a fusion protein of viral p54, which is an early/late protein that mainly accumulates at the viral replication sites (Rodriguez et al., 1994; Hernaez et al., 2006). ASFV viral stocks were propagated and titrated by plaque assay in Vero cells, as previously described (Enjuanes et al., 1976). When using the recombinant viruses BPP30GFP or B54GFP, green fluorescent plaques were observed 4 days after infection under the fluorescence microscope. ASFV stocks were partially purified using a sucrose cushion (40%) in PBS at 68,000 × g for 50 min at 4°C and were further used at a multiplicity of infection (MOI) of 1 unless otherwise indicated.

### 2.3 Generation of stable cell lines

To generate Vero cells deficient in VPS4A or ALIX, we used the commercial lentiviral vector containing shRNA to interfere with VPS4A (TRCN000012998) or ALIX (TRCN0000343595), respectively, obtained from Merck (Darmstadt, Germany). A TRC1 pLKO.1-puro empty shRNA was used as control. Lentiviral particles were produced by transfection of HEK293T cells (seeded in 60-mm dishes) with 5 μg of lentiviral vectors, 3 μg of the human immunodeficiency virus (HIV) gag-pol expressing plasmid, and 3 μg of vesicular stomatitis virus-G glycoprotein using Lipofectamine 2000 (Life Technologies, Carlsbad, CA, USA). Supernatants containing the lentivirus were harvested at 48 and 72 h post transfection, 0.45 μm-filtered, and used to transduce Vero cells, which were subsequently selected for puromycin resistance (Life Technologies, 10 μg/mL).

### 2.4 Antibodies

The following rabbit antibodies were used: VPS4A (H-165) (SCBT, sc-32922), Lamp-1 (Abcam, ab24170), ALIX (Abcam, ab88388), ALIX (Proteintech, 12422-1-AP), or HRS (CST, #14346). Mouse monoclonal antibodies used were as follows: ALIX (Q19) (sc-49268), EEA1 (BD Biosciences, 610457), CD63 (H5C6) (Novus Biologicals, NBP2-42225), p150 (Ingenasa, 17AH2), p72 (Ingenasa,1BC11), p72 (Ingenasa, 18BG3), p30 (a gift from J.M. Escribano, Algenex, Madrid, Spain), or LBPA (6C4) (Echelon Biosciences, Z-SLBPA). ALIX (Q19) (SCBT, sc-9268) was the goat antibody used.

### 2.5 Flow cytometry

Vero cells were infected with recombinant ASFV BPP30GFP or B54GFP at a MOI of 1 pfu/cell for 16 h. Cells were washed with PBS, harvested with Trypsin-EDTA (Gibco, Gaithersburg, MD, USA), and then washed and collected with flow cytometry buffer (PBS, 0.01% sodium azide, and 0.1% bovine serum albumin). In order to determine the percentage of infected cells per condition (infection efficiency), 10,000 cells per time point were scored using a FACS Canto II flow cytometer (BD Sciences, Franklin Lakes, NJ, USA) and analyzed using the FlowJo software. Infected cell percentages obtained were normalized to values found in control samples.

### 2.6 Western blot analysis

Cells were seeded in six-well plates and infected with ASFV at a MOI of 1 pfu/cell. Protein lysates were separated based on electrophoretic mobility in sodium dodecyl sulfate polyacrylamide gels (SDS-PAGE) under reducing conditions and transferred to nitrocellulose membranes (Amersham Biosciences, Amersham, UK). Membranes were blocked with 5% skimmed milk powder in PBS−0.05% Tween-20 (Sigma-Aldrich, Saint Louis, MO, USA) for 1 h and further incubated with primary antibody at 4°C overnight. Finally, immunoblot was incubated with suitable horseradish peroxidase-conjugated secondary antibodies for 1 h at room temperature. Protein expression was analyzed using the molecular imager Chemidoc XRS plus Imaging System. Bands were quantified by densitometry and normalized using the Image Lab software (Bio-Rad, Hercules, CA, USA).

### 2.7 Immunofluorescence

Vero cells were seeded at a variable density onto 12-mm glass coverslips in 24-well plates before infection. Then, cells were washed with PBS and fixed with 4% paraformaldehyde (PFA) for 15 min. After washing with PBS, cells were permeabilized with 0.1% Triton X-100 in PBS for 10 min. Then, coverslips were washed with PBS and incubated in 2% bovine serum albumin (BSA, Sigma) diluted in PBS for 1 h. Slides were then incubated at room temperature for 1 h in primary antibody diluted in PBS–BSA 1%. Appropriate secondary antibodies conjugated to either Alexa Fluor-488 or -594 (Thermo Fisher, Waltham, MA, USA) were used and cell nuclei were detected with TO-PRO-3 (Thermo Fisher Scientific). Coverslips were mounted on glass slides using ProLong Gold (Thermo Fisher Scientific). Cells were visualized using a TCS SPE confocal microscope (Leica) and image acquisition was performed with a Leica Application Suite Advanced Fluorescence software (LAS AF).

### 2.8 Immunofluorescence quantitative analysis

Fluorescence labeling of ALIX antibody was measured using ImageJ 1.53c (NIH). Specific ROIs (regions of interest) were designed for 20 cells per condition, and cytoplasmic fluorescence signal above a certain threshold (adjusted as background) was quantified. Results were expressed as the average of intensity and, relative to values detected in control samples, arbitrarily assigned a value of one.

To quantify the amount of LBPA accumulated close to the viral factory, we used the ImageJ plug-in “Fluorescence Ratio” kindly provided by Dr. Sánchez Sorzano (CNB, Madrid, Spain) (Rincon et al., 2011) that allows us to obtain the fluorescence intensity detected at a particular area of the cell related to other areas. The plugin takes into account the fluorescence provided by the background. In our experiment, we computed the ratio between the fluorescence at the viral factory in the perinuclear region and the total cytoplasm of each cell. We measured the intensity in 30 individual cells per condition (MOCK or infected).

### 2.9 Virus titration

Vero cells were infected with Ba71V or BPP30GFP at a MOI of 1 pfu/cell. After 24 h or 48 h of infection, total viruses from cell lysates and supernatants were collected and titrated by plaque assay in triplicate samples on monolayers of Vero cells. Intracellular virus titers were obtained by repeatedly freezing and thawing infected cells. Vero cells were infected with 10-fold serial dilutions from samples and viral adsorption lasted 90 min in 2% FBS at 37°C. The viral inoculum was then removed and a 1:1 ratio of 2% low-melting-point agarose and complete 2X EMEM was added. Plaque visualization was possible at 10 days after staining with violet crystal. When using the recombinant BPP30GFP, green fluorescent plaques were observed 4 days after infection.

### 2.10 Quantitative real-time PCR

DNA from Vero cells infected with ASFV at an MOI of 1 pfu/cell for 16 h was purified using the DNAeasy blood and tissue kit (Qiagen) following the manufacturer’s protocol. DNA concentration was measured using a Nanodrop spectrophotometer. The qPCR assay amplifies a region of the p72 viral gene, as described previously (King et al., 2003). The amplification mixture was 200 ng of DNA template added to a final reaction mixture of 20 μL containing 50 pmol sense primers, 50 pmol anti-sense primer, 5 pmol of probe, and 10 μL of Premix Ex Taq (2×) (Takara Bio, Shiga, Japan). Each sample was included in triplicate and values were normalized to standard positive controls. Reactions were performed using the ABI 7500 Fast Real-Time PCR System (Applied Biosystems) with the following parameters: 94°C for 10 min and 45 cycles of 94°C for 10 s and 58°C for 60 s.

### 2.11 RNA extraction and quantitative PCR analysis

RNA was extracted from Vero cells grown in six-well plates using the RNeasy RNA extraction kit (Qiagen, Hilden, Germany) according to the manufacturer’s protocol. For retrotranscription, a QuantiTect Reverse Transcription kit (Qiagen) was used to synthesize cDNA, also following the manufacturer’s protocol. cDNA (250 ng) was the template for real-time PCR using the QuantiTect SYBR Green PCR Kit (Qiagen). Reactions were performed using the ABI 7500 Fast Real-Time PCR System (Applied Biosystems, Waltham, MA, USA). Expression of VPS4A was analyzed by using Hs_VPS4A_1_SGQuantiTect Primer Assay QT00022029 (Qiagen) and normalized to an internal control (18S ribosome subunit) to yield the fold expression.

### 2.12 Statistical analysis

The experimental data were analyzed by one-way ANOVA by GraphPad Prism 6 software. For multiple comparisons, Bonferroni’s correction was applied. When comparing two sample means, an unpaired Student’s *t*‐test was performed. Values were expressed in bar graphs as mean ± SD of at least three independent experiments unless otherwise noted. A *p* < 0.05 was considered as statistically significant.

## 3 Results

### 3.1 Role of VPS4A in ASFV infection

**Figure 1:**
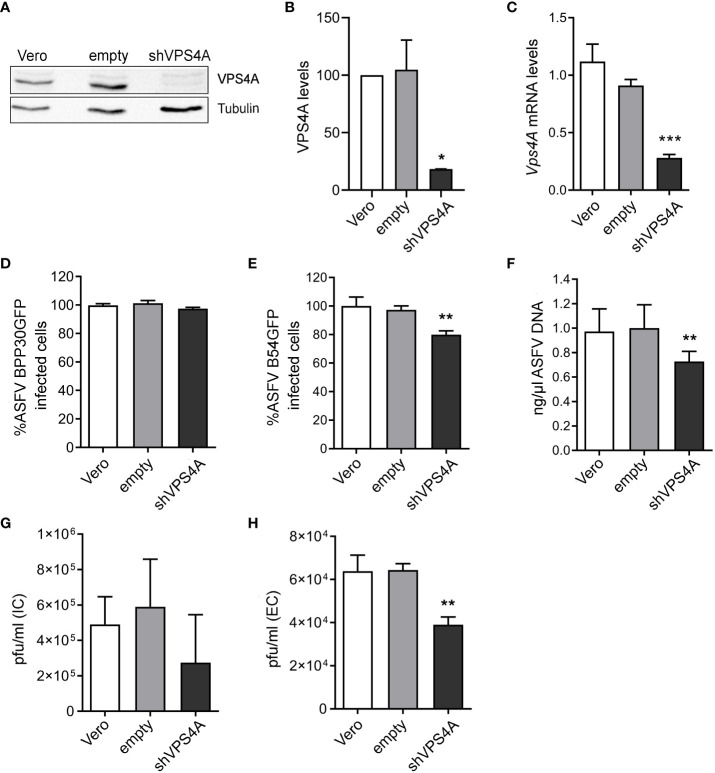
Impact of VPS4A silencing in several infection parameters. **(A)** Representative immunoblot of VPS4A in Vero cells transduced with lentiviral particles encoding VPS4A shRNA (shVPS4A) or empty shRNA (empty) and wild-type Vero cells (Vero) as controls. **(B)** Densitometric analysis of VPS4A expression in WB using alpha-tubulin as loading control and compared with empty values. **(C)** VPS4A mRNA levels analyzed by qPCR using VPS4A-specific primers. Ribosomal protein 18S was used as control. Data are presented as a fold compared to empty cells. **(D)** Infectivity of BPP30GFP (1 pfu/mL) in Vero cells at 16 hpi measured by flow cytometry (GFP fluorescence percentage relative to empty control). **(E)** Percentage of B54GFP-infected cells (GFP fluorescence relative to empty control) at 16 hpi in Vero cells. **(F)** Quantification of ASFV genome copy number by real-time PCR 16 hpi and normalized to empty values. Data are presented as a fold and normalized to empty values. **(G)** Virus titration by plaque assay of intracellular (IC) virions generated in wild-type, empty, or shVPS4A Vero cells infected with BPP30GFP at a MOI of 1 pfu/mL for 24 (h) **(H)** Virus titration by plaque assay of extracellular (EC) virions generated in wild-type, empty, or shVPS4A Vero cells infected with BPP30GFP at a MOI of 1 pfu/mL for 24 hpi. Statistically significant differences are indicated by asterisks (*p < 0.05; **p < 0.01; ***p < 0.001).

To determine the impact of the ESCRT complex on ASFV infection, we silenced the expression of VPS4A using shRNA as described above. The reduction of VPS4A expression levels was confirmed by Western blot (**Figures 1A, B**) and RT-qPCR (**Figure 1C**). After assessing the silencing, we were interested in identifying whether VPS4A interfered in ASFV infectivity. Wild-type, empty shRNA (empty) and VPS4A shRNA (shVPS4A) Vero cells were infected with ASFV BPP30GFP, a recombinant virus that expresses the GFP protein under the early viral protein promoter p30. No effect was detected at early stages post-infection by flow cytometry using this tagged recombinant virus (**Figure 1D**). However, when cells were infected with recombinant virus B54GFP, expressing GFP as a p54 fusion protein resulted in an inhibition of the infectivity. In fact, the silencing of VPS4A reduced the percentage of fluorescent cells when measured by flow cytometry at 16 h post infection compared to empty cells (**Figure 1E**). In agreement with the result shown in **Figure 1E**, we obtained a significant decrease of ASFV replication in shVPS4A cells analyzed by quantitative PCR (**Figure 1F**). In order to gain knowledge of the impact of VPS4A silencing in the last stage of ASFV infection, wild-type, empty, and shVPS4A Vero cells were infected with BPP30GFP in order to quantify the amount of intracellular virus produced in each condition, and also evaluate the amount of virus released to the extracellular space. After 24 h of infection, intracellular (IC) and extracellular (EC) virus production were analyzed. Cellular pellets and supernatants were collected and titrated by plaque assay in Vero cells as described in *Materials and Methods*. As shown in **Figure 1G**, no significant differences were observed when titrating IC viral production generated in Vero, empty, and shVPS4A Vero cells. Interestingly, the shVPS4A cell line produced less EC viral particles compared to empty Vero cells (**Figure 1H**).

### 3.2 Effect of VPS4A silencing in the redistribution of endosomal membranes during ASFV infection

Previous studies have shown a reorganization of endosomal traffic and endosomal membranes remodeling close to the viral replication site or viral factory (VF) (Cuesta-Geijo et al., 2017) that could be eventually altered under the inhibition of the ESCRT component VPS4A. The ASFV VF is the site where the viral replication takes place, and it is visualized as a perinuclear structure by confocal microscopy.

**Figure 2:**
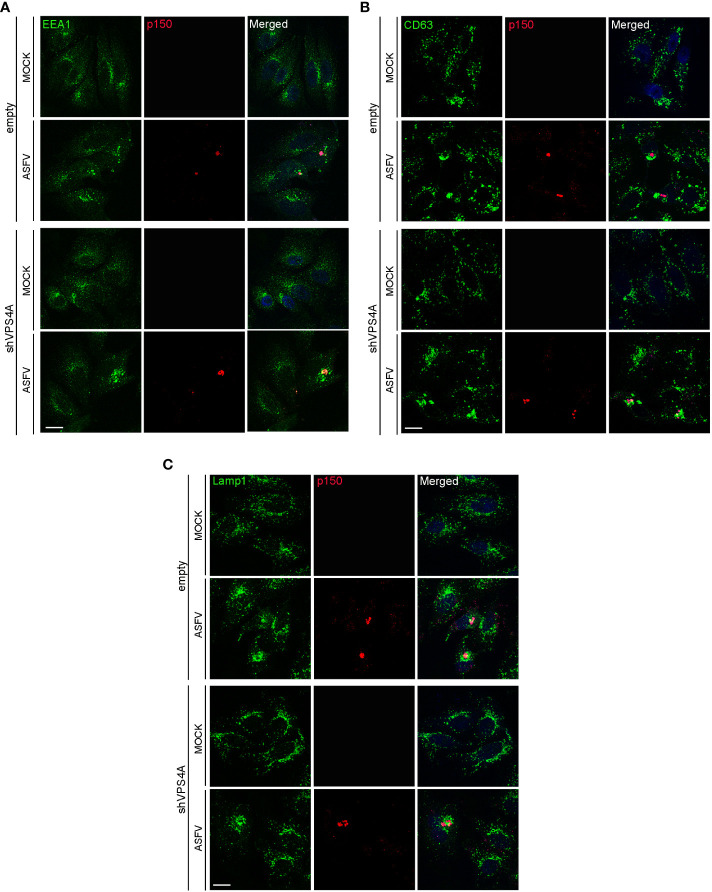
VPS4A and vesicle reorganization at viral factories. Representative confocal images of empty and shVPS4A Vero cells uninfected (MOCK) or infected with Ba71V at a MOI of 1 pfu/cell for 16 (h) After fixation, cells were stained for viral core protein p150 (red), DNA (TO-PRO-3, blue), and vesicle membrane marker EEA1 (green) **(A)**, CD63 (green) **(B)**, or Lamp1 (green) **(C)**. Scale bar: 20 µm.

Empty and shVPS4A Vero cells were infected with ASFV for 16 hpi. Cells were fixed and assayed with immunofluorescence to detect the early endosome marker EEA1, the MVB marker CD63, and the lysosomal marker Lamp1. As previously described, immunofluorescence images showed EEA1-labeled early endosomes around the VF. The VF was recognized by the labeling with the structural viral protein p150 at 16 hpi in infected empty cells (**Figure 2A**). Silencing of VPS4A maintained EEA1 recruitment close to the VFs (**Figure 2A**). In CD63 labeling, we detected a diffuse cytoplasmic localization of MVBs in mock empty cells (**Figure 2B**), while CD63 repositioned close to the VF in infected empty cells. A similar pattern was found in mock- as well as ASFV-infected shVPS4A cells (**Figure 2B**). When visualizing the reorganization of lysosomes in the absence of VPS4A, no alteration was observed either in mock-infected or in ASFV-infected cells, when comparing empty and shVPS4A cell lines (**Figure 2C**). In summary, no alterations in the distribution of cellular vesicles were detected in either mock or infected cells under VPS4A silencing conditions.

### 3.3 Impact of VPS4A silencing in ALIX expression during ASFV infection

**Figure 3:**
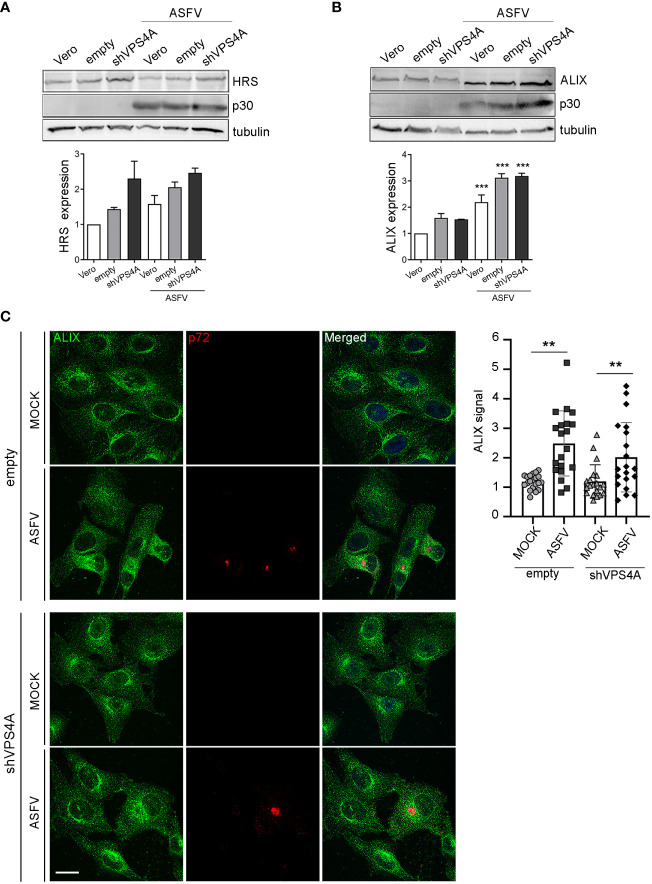
ALIX expression in cells lacking VPS4A. **(A, B)** Representative immunoblot and densitometric analysis of HRS expression **(A)** or ALIX expression **(B)** in MOCK and infected samples harvested at 16 hpi (MOI of 1 pfu/cell) for wild-type Vero cells (Vero), empty shRNA (empty), or VPS4A shRNA (VPS4A). ASFV infection was detected by using viral protein p30. Alpha-tubulin was used as loading control. Data are presented as mean ± SD of densitometry values from two independent experiments. **(C)** Illustrative confocal images of empty (upper panel) and shVPS4A (lower panel) Vero cells stained with ALIX (green). Viral factories were detected with the antibody against p72 (red) and DNA with TO-PRO-3 (blue). Quantification of ALIX staining in MOCK or infected (ASFV) in empty and shVPS4A cell lines. Each dot represents a single cell (*n* = 20). Scale bar: 20 µm. Statistically significant differences are indicated by asterisks (**p < 0.01; ***p < 0.001).

To gain insight into how silencing VPS4A could affect other ESCRT components and ASFV infection, we focus on HRS (ESCRT-0) and the adaptor protein ALIX, a crucial player for several virus models to pursue a successful infection (Le Blanc et al., 2005; Munshi et al., 2007; Pattanakitsakul et al., 2010; Pasqual et al., 2011; Jiang et al., 2020). This protein participates in actin assembly and membrane remodeling processes settled by ESCRT-III (McCullough et al., 2018; Qiu et al., 2022), which are relevant for several viruses (Lin et al., 2022). We infected wild-type, empty, and shVPS4A Vero cells with ASFV at a MOI of 1 pfu/cell and they were harvested at 16 hpi. Western blot analysis showed a tendency towards the increase of ESCRT-0 component HRS in silenced VPS4A cells, slightly dependent on ASFV infection (**Figure 3A**). Similar data were observed in Vero wild-type cells, used as control. When studying ALIX expression, we observed an increase in cells infected with ASFV at 16 hpi compared to mock cells. This increment was independent of the silencing of VPS4A (**Figure 3B**). Finally, we monitored the pattern of ALIX during ASFV infection in both empty and shVPS4A Vero cells by immunofluorescence (**Figure 3C**). In agreement with immunoblot experiments, infected shVPS4A cells (lower panel, **Figure 3C**) showed a higher ALIX labeling than mock shVPS4A cells (lower panel, **Figure 3C**). Similar results were obtained from empty cells (upper panel, **Figure 3C**). ALIX distribution around viral factory (labeled with p72) was detected in both cell lines (**Figure 3C**). These results point out that HRS is dependent on VPS4A silencing but weakly on ASFV infection. Conversely, ALIX overexpression occurs under infection conditions and it is not related to VPS4A depletion.

### 3.4 ASFV replication and virus production are impacted by ALIX knockdown

**Figure 4:**
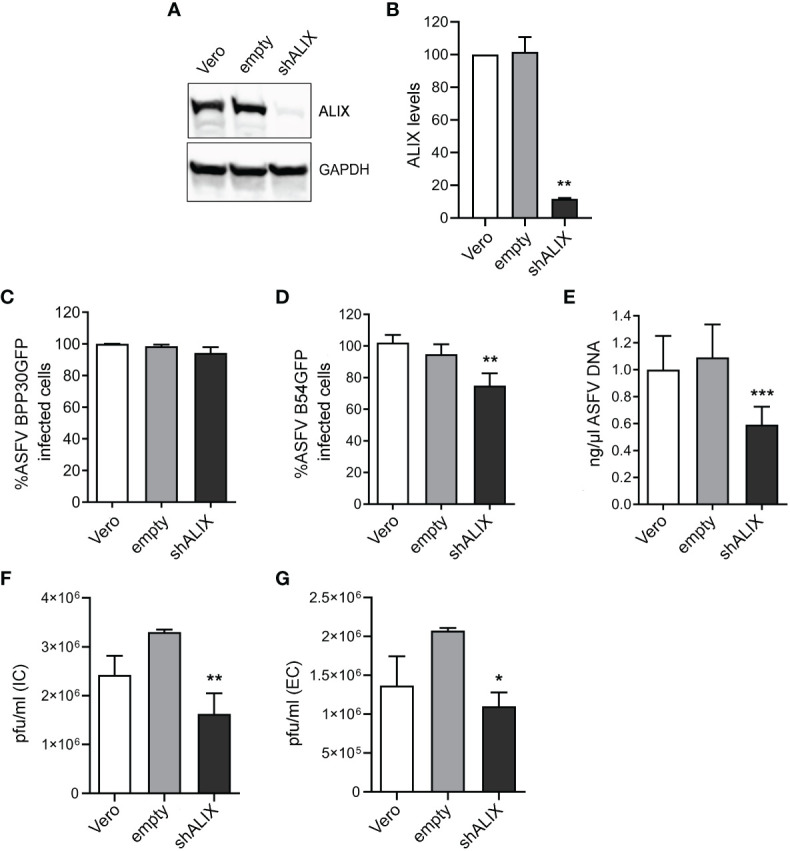
ALIX depletion affects ASFV infection. **(A, B)** Representative immunoblot of ALIX in Vero cells transduced with lentiviral particles encoding ALIX shRNA (shVPS4A) or empty shRNA (empty) and wild-type Vero cells (Vero) as controls. **(B)** Densitometric analysis of ALIX expression in WB using GAPDH as loading control and compared with empty values. Data are presented as mean ± SD of densitometry values from two independent experiments. **(C, D)** Flow cytometry of wild-type, empty, and shALIX Vero cells infected with fluorescent recombinant ASFV BPP30GFP **(C)** or B54GFP **(D)** at a MOI of 1 pfu/mL for 16 hpi. **(E)** Quantification of ASFV DNA by qPCR at 16 hpi. Data are presented as a fold and normalized to empty values. **(F, G)** Virus titration by plaque assay of intracellular (IC) and extracellular (EC) virions generated in wild-type, empty, or shALIX Vero cells infected with BPP30GFP at a MOI of 0.1 pfu/mL for 48 h, respectively. Statistically significant differences are indicated by asterisks (*p < 0.05; **p < 0.01; ***p < 0.001).

Using a validated shRNA targeting ALIX, we conducted RNA silencing of the protein in Vero cells to investigate the function of ALIX in ASFV infection. Immunoblotting of cell lysates revealed that gene knockdown reduced protein expression of ALIX at 10% after silencing (**Figures 4A, B**).

After that, cells were infected with ASFV. As observed in **Figure 4C**, viral internalization of ASFV BPP30GFP was not affected, since early viral protein expression p30 was unaltered after ALIX knockdown, detected through GFP by flow cytometry. Conversely, the late viral protein p54 decreased under silencing conditions (**Figure 4D**). Indeed, we confirmed this by observing a significant decrease of ASFV replication in shALIX Vero cells by using qPCR (**Figure 4E**).

To further assess a function for ALIX at later stages at the viral cycle, we analyzed the viral production by the plaque assay. In agreement with viral replication results, silencing of ALIX triggered a 1.5 log reduction in intracellular viral titer, as well as in extracellular viral titer in shALIX Vero cells compared to empty Vero cells (**Figures 4F, G**).

### 3.5 LBPA plays a role in ASFV infection

**Figure 5:**
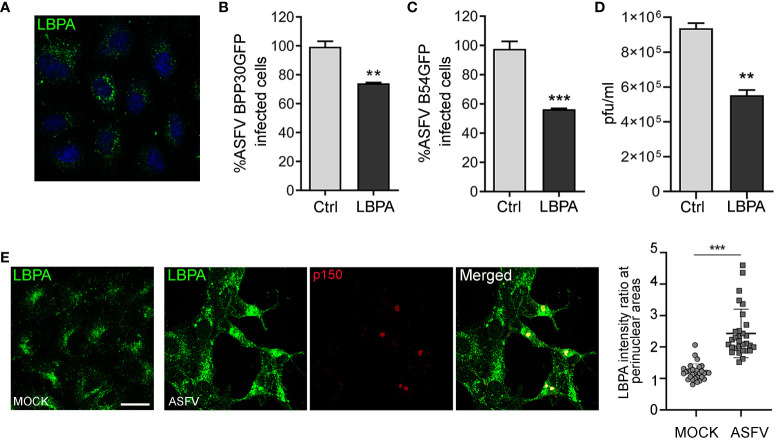
LBPA affects ASFV infection. Vero cells were pre-incubated with no antibody (Ctrl) or 50 µg/mL monoclonal antibody anti-LBPA for 24 (h) **(A)** LBPA internalization was processed for immunofluorescence and analyzed by confocal microscopy **(B, C)** Cells were then infected with recombinant virus BPP30GFP or B54GFP. In treated samples (LBPA), a virus adsorption period (90 min at 37°C) was conducted in the presence of the Ab. After washing out unbound viral particles, Vero cells were incubated at 37°C in fresh medium. **(B)** Infectivity of BPP30GFP (1 pfu/mL) in Vero cells at 6 hpi measured by flow cytometry (GFP fluorescence percentage relative to control). **(C)** Percentage of B54GFP-infected cells (GFP fluorescence relative to empty control) at 16 hpi in Vero cells. **(D)** Total ASFV production quantified by plaque assay of Vero cells untreated (Ctrl) or incubated with LBPA for 24 h and infected with ASFV. **(E)** Vero cells infected with ASFV for 16 h and staining with anti-LBPA antibody (green) and core protein p150 (red). Graph shows the quantification of LBPA staining in the perinuclear area related to the cytoplasm in MOCK and infected Vero cells. Each dot in the graph represents the ratio of fluorescence measured at the perinuclear region related to the cytoplasm per cell (*n* = 30 per condition). Scale bar: 20 µm. Statistically significant differences are indicated by asterisks (**p < 0.01; ***p < 0.001).

Enhanced expression of ALIX and its role in ASFV infection lead us to study the possible involvement of lysobisphosphatidic acid (LBPA), an unconventional lipid that interacts with ALIX, in regulating endosomal homeostasis and cholesterol efflux from the LE, which are crucial for ASFV infection.(McCauliff et al., 2019). To test the effect of LBPA in ASFV entry and infection, we used the blocking monoclonal antibody LBPA 6C4 that inhibits LBPA activity (Kobayashi et al., 1998) and LBPA–ALIX interaction (Pattanakitsakul et al., 2010). We first tested the correct LBPA antibody internalization (**Figure 5A**). Thus, Vero cells were incubated with anti-LBPA antibody for 24 h and subsequently infected with BPP30GFP (MOI of 1 pfu/cell). Pre-incubation with anti-LBPA antibody resulted in a significant decrease of ASFV entry at 6 hpi, as observed when we analyzed the percentage of GFP-positive cells by flow cytometry (**Figure 5B**). In concordance with the previous results, we detected a higher impact of LBPA blocking at later stages of the infection, reaching almost 50% of inhibition in Vero cells treated and infected with B54GFP (**Figure 5C**). Our observations of reduced ASFV infectivity under LBPA antibody treatment was further supported by a decrease in total viral production (**Figure 5D**). Finally, we illustrated the pattern of endogenous LBPA by staining Vero-infected cells at 16 hpi and processing them by confocal microscopy. As shown in **Figure 5E**, LBPA staining distributed with the same pattern as late endosomes, around the viral factories in infected cells colocalizing with p150-labeled replication sites. Quantification of the amount of LBPA clustered around the viral factory (determined as a ratio fluorescence intensity close to the VF/total fluorescence) showed that LBPA accumulates specifically around VFs compared to the cytoplasm in infected cells (ASFV). In uninfected cells (MOCK), LBPA signal distribution was more homogeneous in the perinuclear region and cytoplasm (**Figure 5E**).

## 4 Discussion

Increasing lines of evidence indicate that several viruses usurp the cellular ESCRT machinery at several infection stages (Votteler and Sundquist, 2013; Weissenhorn et al., 2013; Scourfield and Martin-Serrano, 2017). However, it is still unknown whether ESCRT proteins could participate in ASFV infection.

ESCRT-III functions are coordinated by the AAA-ATPase VPS4, which regulates the exchange of subunits during ESCRT-III polymerization and disassembly upon membrane remodeling and membrane scission (Davies et al., 2010; Adell and Teis, 2011; Adell et al., 2017; Pfitzner et al., 2020; Vietri et al., 2020). In addition, several virus families exploit VPS4 for a successful infection. This protein has a relevant function in the generation of the virion envelope of herpesvirus in the nucleus of infected cells (Crump et al., 2007; Pawliczek and Crump, 2009; Yadav et al., 2017), and alterations in this protein impair the egress of viral particles through the inner nuclear membrane (Lee et al., 2012; Arii et al., 2018). Classical swine fever virus, Echovirus 1, or Arenavirus also require VPS4 function for their infection (Karjalainen et al., 2011; Pasqual et al., 2011; Liu et al., 2022), and recently, the relevance for VPS4 in HIV budding has been shown (Harel et al., 2022). Nevertheless, in other enveloped viruses, VPS4 has merely an ESCRT-III recycling function (Diaz et al., 2015), or it is needless, as shown for flavivirus replication (Tabata et al., 2016). Consistent with a central role for VPS4 in several viral infections, we proceed to evaluate the impact of silencing VPS4A in ASFV infection. ASFV replication was affected when silencing VPS4A expression in cells, mainly at later stages of the viral cycle. Changes in the level of VPS4A did not impair ASFV infectivity at early stages; therefore, VPS4A silencing suggests an implication for a later stage in viral infection. It could be possible that VPS4A assists the viral progeny assembly during the virion membrane acquisition, as occurs with Vaccinia virus, a closely related nucleocytoplasmic DNA virus (Huttunen et al., 2021). In fact, VPS4A silencing reduced extracellular virus production; hence, this would suggest a potential role for this ESCRT protein in ASFV budding, similarly to HIV-1 (Kieffer et al., 2008; Harel et al., 2022).

The redistribution of membranes, analyzed through endosomal and lysosomal markers, remained altered in infected cells, without any evident effect in the redistribution of vesicle membranes under VPS4A silencing. A plausible reason for this finding could be a functional redundancy between VPS4A and VPS4B that would compensate for the lack of VPS4A at particular infection stages, as has previously been described for other ESCRT components such as CHMP2 or CHMP4 families in flavivirus infection (Tabata et al., 2016).

The ESCRT-0 component HRS initially guides ubiquitinated membrane proteins from early endosomes into ILVs of the MVBs (Vietri et al., 2020). ASFV weakly increased HRS expression; however, virions that traffic through endosomes would occur independently of ILVs, since ILVs have a smaller size than ASFV virions and require the acidic pH of LEs to decapsidate (Cuesta-Geijo et al., 2012). Thus, this requirement of HRS in ASFV infection is not yet well understood.

The accessory ESCRT protein ALIX exerts multiple functions, including endosomal homeostasis in cooperation with other ESCRT components (Gupta et al., 2020; Tran et al., 2022). Contrarily to HRS, ASFV-infected cells overexpressed and relocated ALIX around the viral factory independently of VPS4A silencing. As ALIX was recruited to the viral factory, we hypothesized that the protein could have a role during ASFV infection, supporting later stages during the viral cycle as replication, viral production, or egress, as occurs in flavivirus infection (Tran et al., 2022), HIV-1 (Fujii et al., 2009), or classical swine fever infection (Liu et al., 2022). This assumption was supported by a reduction of p54 expression and viral copy number during replication in cells lacking ALIX. According to these data, viral production was altered, as indicated by the reduction in intracellular and extracellular viral titer. We could speculate about the involvement of ALIX in viral morphogenesis and viral egress since intracellular and extracellular titers were affected, or it could be also a consequence of a defective viral replication. Thereby, since replication is affected and intracellular and extracellular plaque assay results are comparable, with no severe effects in titer, ALIX could support viral replication, but it has a modest role in viral production and budding. Further studies need to be conducted to clarify this issue.

Besides its role with ESCRT, ALIX controls ILV formation in the MVB and LE dynamics through the interaction with LBPA by an exposed site in the ALIX Bro1 domain in a Ca^2+^-dependent manner (Williams and Urbe, 2007; Bissig and Gruenberg, 2014). Interestingly, it has been recently reported that ASFV triggers the activation of calcium-signaling pathways in endosomes (Galindo et al., 2021).

LBPA is specifically located particularly in the inner leaflet of late endosomes and a variety of viruses, such as dengue virus or vesicular stomatitis virus, take advantage of this molecule for membrane fusion at entry. In fact, pretreatment with anti-LBPA antibody reduces dengue and Arenavirus infection (Pattanakitsakul et al., 2010; Zaitseva et al., 2010; Roth and Whittaker, 2011). We impaired LBPA function by preincubating cells with a blocking function monoclonal antibody against LBPA prior to ASFV infection, which affects endosomal/MVBs homeostasis or back fusion events altering the cholesterol efflux necessary for ASFV infection (Bissig and Gruenberg, 2014; Gruenberg, 2020; Cuesta-Geijo et al., 2022). Our data support the idea that LBPA blocking treatment prior to infection reduces ASFV infectivity at early and late stages post-infection, thus affecting virions trafficking through endosomes, replication, and also viral production. This indicates how LBPA controls ASFV infectivity and viral production, probably by affecting cholesterol efflux, in an ALIX unrelated process, since ALIX knockdown did not alter early protein expression during ASFV infection.

Cholesterol traffic and the main intracellular cholesterol transporters NPC1 and 2 are relevant in ASFV infectivity and replication (Cuesta-Geijo et al., 2022). Thus, the direct interaction between NPC2 and LBPA to regulate cholesterol efflux could possibly affect earlier stages of ASFV infection (McCauliff et al., 2019). Furthermore, LBPA is redistributed to perinuclear locations, where ASFV replication occurs, supporting a function in viral infection at later stages.

Interestingly, recent works have shown that LBPA is important for the life cycle of SARS-CoV-2 (Carriere et al., 2020; Luquain-Costaz et al., 2020), but more studies are required to unveil its mechanism of action in the infection. Our results would support that ASFV needs an efficient cholesterol transport that depends on LBPA and NPC proteins from early steps of the infection, while the LBPA–ALIX interaction, related to ILV formation and back fusion, would remain in the background, since ASF virions fuse with the LE (Cuesta-Geijo et al., 2012; Hernaez et al., 2016).

In summary, our results point out for the first time the involvement of ESCRT-related proteins such as VPS4A, ALIX, and the unconventional phospholipid LBPA in ASFV infection. These molecules are tightly linked to the maintenance of endocytic pathway homeostasis, and their functions impact the infectivity and replication of ASFV. The results presented constitute an interesting opening for addressing the specific function of these proteins in future works, unveiling potential new therapeutic molecules with a putative antiviral activity.

## Data availability statement

The original contributions presented in the study are included in the article/supplementary material. Further inquiries can be directed to the corresponding author.

## Author contributions

CA and MC-G contributed to conception and design of the study. B-GL and MC-G conducted experimentation and CA validation. ID and IG conducted the image quantification and formal analysis. CA was responsible for the funding of the project. All authors wrote sections of the manuscript. All authors contributed to manuscript revision and approved the submitted version.
